# *Sema6D* Regulates Zebrafish Vascular Patterning and Motor Neuronal Axon Growth in Spinal Cord

**DOI:** 10.3389/fnmol.2022.854556

**Published:** 2022-04-07

**Authors:** Jiajing Sheng, Jiehuan Xu, Kaixi Geng, Dong Liu

**Affiliations:** ^1^Nantong Laboratory of Development and Diseases, Key Laboratory of Neuroregeneration of Jiangsu and Ministry of Education, School of Life Science, Second Affiliated Hospital, Co-innovation Center of Neuroregeneration, Nantong University, Nantong, China; ^2^Medical School, Nantong University, Nantong, China

**Keywords:** endothelial cells, motor neuronal axon, guidance cues, path finding, zebrafish

## Abstract

Vessels and nerves are closely associated in anatomy as well as functions. Accumulating evidences have demonstrated that axon-guiding signals may affect endothelial cells migration and path finding, which is crucial for the patterning of both the complex vascular network and neural system. However, studies regarding the functional overlap between vascular and neuronal orchestrating are still incomplete. Semaphorin6D (Sema6D) belongs to the Semaphorin family and has been identified as an important regulating factor in diverse biological processes. Its roles in vascular development are still unclear. Here, we confirmed that *sema6D* is enriched in neural system and blood vessels of zebrafish embryos by *in situ* hybridization. Then, the deficiency of *sema6D* caused by specific antisense morpholino-oligonucleotides (MO) led to dramatic path finding defects in both intersegmental vessels (ISVs) and primary motor neurons (PMNs) of spinal cord in zebrafish embryos. Furthermore, these defective phenotypes were confirmed in F0 generation of *sema6D* knockouts and rescue experiments by overexpression of *sema6D* mRNA in *sema6D* morphants. These data collectively indicate that *sema6D* regulates zebrafish vascular patterning and motor neuronal axon growth in the spinal cord, which might be of great therapeutical use to regulate vessel and nerve guidance in the relevant diseases that affect both systems.

## Introduction

Vascular formation is an early and essential process during the development of vertebrate embryos (Nikolova and Lammert, 2003). Vascular endothelial cells assemble into tubes and then couple into an initial vascular pattern. The stereotypical wiring pattern of vessels is essential for its functions and its abnormality is inextricably related to a wide range of diseases, including atherosclerosis, hypertension, aneurysms, diabetic retinopathy, aneurysms, and tumor (Carmeliet, 2003). Although studies on angiogenesis have made extraordinary progress, the detailed regulatory mechanisms of vascular patterning remain to be fully explored (Patan, 2000; Makanya et al., 2009; Uccelli et al., 2019). Vessels and nerves are closely associated in anatomy as well as functions. Increasing reports have demonstrated that axon-guiding signals may affect endothelial cells migration and path finding, which is crucial for the development of both the vascular and the nervous systems. However, studies regarding the functional overlap between vascular and neuronal pathways are still limited and need advanced research (Luo et al., 1993; Kolodkin and Tessier-Lavigne, 2011).

Semaphorins that are expressed in most organs and tissues were originally described as axon guidance in the hard wiring of the nervous systems (Bussolino et al., 2006; Suchting et al., 2006). However, increasing reports attested to the significance of Semaphorinsin the development and regulation outside the neuron system (Neufeld and Kessler, 2008; Capparuccia and Tamagnone, 2009). Members of Semaphorins have been found to regulate the cells motility during the development of nervous (Sema3A, 3F, 4D, 6C, 7A), immune (Sema4D), reproductive (Sema3), cancer (Sema3A, 4D) and vascular systems (Sema3A, 3E, 4D; Gherardi et al., 2004; Gu et al., 2005; Sakurai et al., 2012; Acker et al., 2018; Elder et al., 2018; Ferreira et al., 2018). Semaphorins6D (Sema6D) is a member of Semaphorin family and has been identified as an important regulating factor in the development of the spinal cord, optic nerve, heart, and tumor (Toyofuku et al., 2004a; Yazdani and Terman, 2006; Yoshida et al., 2006; Leslie et al., 2011; Peng et al., 2016). Additionally, Sema6D controls endothelial cell migration during heart development, which may imply its potential role in vascular development (Toyofuku et al., 2004a). However, there is so far no data proving the pro-angiogenic effects of Sema6D yet.

Zebrafish intersegmental vessels (ISVs) are an excellent system to trace cell behavior and morphology *in vivo* by expressing the fluorescent protein in transparent embryos (Ellertsdóttir et al., 2010). To further explore the function of *sema6D* in vertebrate development, we investigated the sequence homology, embryonic expression pattern, and function of *sema6D* in zebrafish. The results present in this study have shown that *sema6D* regulates zebrafish vascular patterning and motor neuronal axon growth in spinal cord. The findings might serve as a ground work for studying the molecular mechanism of neurovascular communication, and might be of therapeutically use to guide vessels and nerves in the relevant diseases that affect both systems.

## Materials and Methods

### Zebrafish Husbandry and Breeding

The study was conducted conforming to the local institutional laws and the Chinese law for the protection of animals. All adult zebrafish (*Dario rerio*) were maintained under standard conditions in accordance with our previous protocols (Huang et al., 2013; Xu et al., 2014). The *AB/WT*, *Tg(kdrl:ras-mCherry)* and *Tg(mnx1:EGFP)* zebrafish used in this article have been described previously (Nicoli et al., 2012; Jao et al., 2013). Zebrafish embryos after 24 hpf were treated with 0.2 mM 1-phenyl-2-thio-urea to prevent pigment formation.

### Phylogenetic Analysis

The zebrafish *sema6D* protein sequences (NP_998164.3) and zebrafish *sema6dl* sequences (XP_005173705.1) together with other nine species, including *Bos taurus* (XP_024853302.1), *Gallus gallus* (XP_040562440.1), *Homo sapiens* (XP_024305841.1), *Mus musculus* (XP_030105194.1), *Oryctolaguscuniculus* (XP_017203544.1), *Oryziaslatipes* (XP_011486693.1), *Rattus norvegicus* (XP_038960910.1), *Sus scrofa* (XP_020951762.1), and *Xenopus tropicalis* (XP_031753477.1) were got from NCBI and used for phylogenetic analysis. The alignments of these sequences were constructed by MEGA7and the phylogenetic tree was constructed by ML.

### RNA Isolation, Reverse Transcription (RT), Polymerase Chain Reaction (PCR), Quantitative RT-PCR, and RNA Probe Transcription

Total RNA of zebrafish embryos at various stages was extracted with TRizol according to the manufacturer’s instruction (Invitrogen, Waltham, MA, USA) and genomic contaminations were removed by DNaseI. Quantity of isolated RNA was verified using gel electrophoresis and Nanodrop, followed by cDNA synthesis using Transcriptor First Strand cDNA Synthesis Kit (Roche), and then was stored at −20°C.

Primers for PCR were designed by the Primer Premier six software and listed as following:

*sema6D*-QF: 5’-CCTCCTCCTATTCCTCTTCTGTT-3’;

*sema6D*-QR: 5’-ACTACGGTGCGGTTCTTATGA-3’;

*ef1a*–QF: 5’-GAGTTGTGCCGTACATCAG-3’;

*ef1a*–QR: 5’-CGTGAGAGTACATGGTCATG-3’.

Quantitative RT-PCR was conducted in a total 20 μl reaction volume with 10 μl SYBR premix (TIANGEN). The relative RNA amounts were calculated with the comparative CT (2-DDCT) method and normalized with elongation factor 1-alpha (ef1a) as the reference. Whole-mount *in situ* hybridization (WISH) with antisense RNA probes was synthesized as described previously (Wang et al., 2016). The cDNA fragments used for *sema6D* RNA probe transcription as templates were amplified using the forward primer 5’-CGACGGCTATCACTTCACTCT-3’and reverse primer 5’-TGGAACATTCTGACGGCTCTT-3’. Then a 548bp sequence of *sema6D* was inserted into pGEM-T-easy vector. Digxigenin (DIG)-labeled sense and antisense probes were performed from the linearized pGEM-T-easy plasmids using the DIG RNA Labeling Kit (Roche).

### Whole Mount *In situ* Hybridization

Whole-mount *in situ* hybridization (WISH) was performed according to our previous procedures (Huang et al., 2013). Digoxigenin-labeled antisense probes were constructed as described above. Zebrafish embryos without pigment at different developmental stages were collected and fixed with 4% PFA overnight at 4°C. After incubated with the probe overnight, an alkaline phosphatase-conjugated antibody against digoxigenin and AP-substrate NBT/BCIP solution (Roche, Switzerland) was used to detect the digoxigenin-labeled RNA probe.

### Morpholino and mRNA Injections

Splicing-blocking Morpholino (5’- TGTGAGCTGAGTGAATGCAGACCT -3’) that was specific for *sema6D* gene was synthesized by Gene Tools. The Morpholino was diluted to 0.3 mM with RNase-free water. The single cell stage embryos of *Tg(kdrl:ras-mCherry)* and *Tg(mnx1:EGFP)* zebrafish were obtained for microinjections as described previously (Wang et al., 2016). Then, the embryos were raised in E3 medium at 28.5°C for following imaging.

### sgRNA/Cas9 mRNA Synthesis and Injections

Cas9 mRNA was obtained by *in vitro* transcription with the linearized plasmid pXT7-Cas9 according to the procedure previously described (Nakayama et al., 2013). The *sema6D* guide RNA (gRNA; 5’-GGCGTGGCAGAAGTAATGAGTGG-3’) was designed and synthesized followed the previously reported (Chang et al., 2013). Transgenic zebrafish lines *Tg(kdrl:ras-mCherry)* and *Tg(mnx1:EGFP)* were natural mated to obtain embryos for microinjection. One to two-cell stage zebrafish embryos were injected with 2–3 nl of a solution containing 250 ng/μl Cas9 mRNA and 15 ng/μl sgRNA (Gong et al., 2017). At 72 hpf, 10 zebrafish embryos were randomly collected and mixed for genomic DNA extraction according to the previous methods (Gong et al., 2017). Then, the amplicons from the genomic DNA were cloned into pGEM-T-easy vector and after transformation, 50 clones were selected randomly for sequencing. The inserted fragments were sequenced by using the forward primer 5’-CCTGTGCATATAGATTGTTG-3’ and reverse primer 5’-AAGTCTACAGACAGTAACG-3’. The mutation efficiency of Crispr-Cas9 was then calculated based on the sequencing results (Wu et al., 2018).

### Rescue Experiments

Full-length and truncated coding sequences of *sema6D* were synthesized and inserted into PCS^2+^ vector as templates for *in vitro* transcription. The mRNA synthesis was carried out by using the mMESSAGEmMACHINESp6 Ultra Kit (Ambion) and purified with the MEGAclearTM Transcription Clean-Up Kit (Ambion) before the injection. Finally, 2 nl capped mRNA was co-injected with *sema6D* Mo into one-cell stage embryos. The *fli1a:sema6D* plasmid was constructed by LR recombination as described in the LifetechMultiste Gateway Manual (Life Technologies, Carls-bad, CA, USA). Then, the construct was injected into one cell stage embryos of *Tg(mnx1:EGFP::kdrl:ras-mcherry)* zebrafish for tissue specific rescue experiments (1 ng per embryo).

### Microscopy and Statistical Analysis

After being anesthetized with tricaine, the zebrafish embryos were mounted in 0.8% low melt agarose and then photographed by Leica TCS-SP5 LSM confocal microscope. For the *in situ* hybridization, Photographs were taken using an Olympus stereomicroscope MVX10. Statistical analyses were performed by one-way analysis of variance (ANOVA) and the Mann-Whitney test. Statistical differences were considered significant for *P*-values <0.05.

## Results

### *sema6D* Gene Is Evolutionarily Conserved in Vertebrates

**Figure 1:**
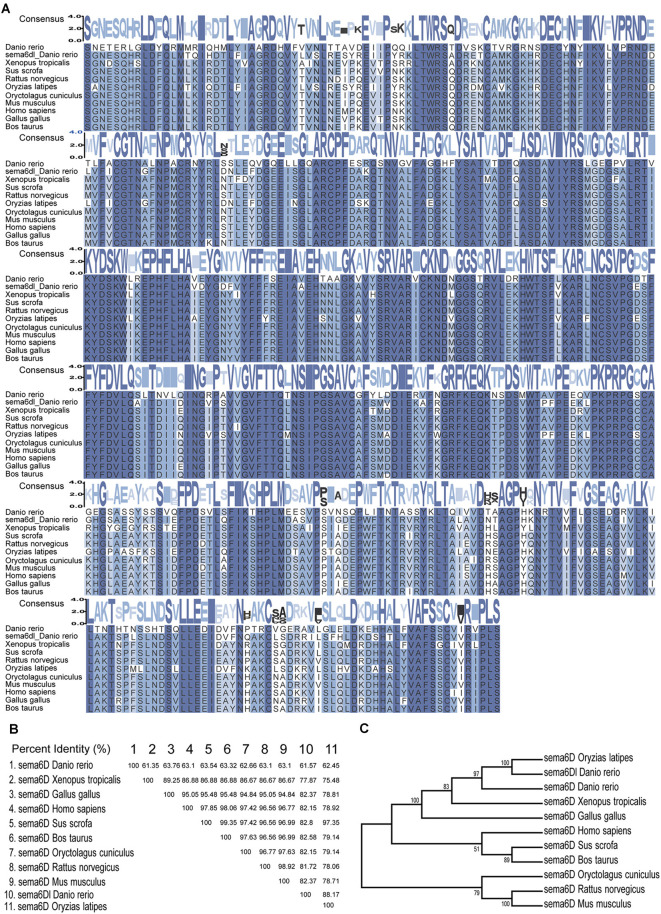
*Sema6D* Is highly conserved during evolution. **(A)** Alignment of the amino acid sequences of the sema domain of *sema6D/sema6Dl* in *Danio rerio*, *Bostaurus, Gallus gallus, Homo sapiens, Musmusculus, Oryctolaguscuniculus, Oryziaslatipes, Rattusnorvegicus, Sus scrofa, and Xenopus tropicalis*. Consensus values indicate the similarity of amino acid sequence, and the greater the similarity. These protein sequences were aligned using MEGA7 software and edited by TBtools. **(B)** The amino acid sequence similarity (%) of sema domain in above species. **(C)** Phylogenetic tree of the sema6D protein.

To analyze the homology of zebrafish *sema6D* with other homologous genes, the multiple alignments and phylogenetic analyses of *sema6D/sema6Dl* were performed with species including *Danio rerio*, *Bostaurus*, *Gallus gallus*, *Homo sapiens*, *Musmusculus*, *Oryctolaguscuniculus*, *Oryziaslatipes*, *Rattusnorvegicus*, *Sus scrofa*, and *Xenopus tropicalis*. As shown in Figures 1A,B, the *sema6D* proteins are significantly conserved during evolution, especially for the sema domain, suggesting their important functions. In addition, zebrafish *sema6D* was clustered in a separate clade with *Oryziaslatipes* and was close to *Xenopus tropicalis* in the phylogenetic tree constructed by complete amino acid sequences of all the *sema6D* proteins above (Figure 1C). Interestingly, the *sema6Dl*, a *sema6D* isoform gene in zebrafish, was clustered with *sema6D* from *Oryziaslatipes*, suggesting it may not be functionally related to *sema6D* in zebrafish.

### Expression of *sema6D* Genes in Zebrafish

**Figure 2:**
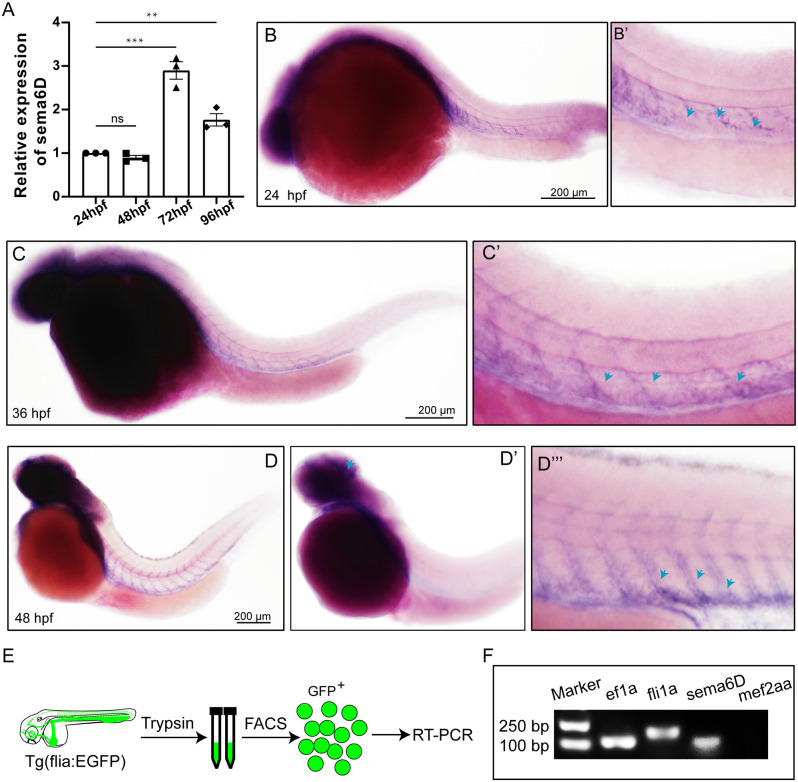
Expression of *sema6D* gene in embryonic zebrafish at different stages. **(A)** QRT-PCR analysis of *sema6D* expression in embryonic zebrafish at different stages (*n* = 3). One-way ANOVA, values with ** and *** above the bars are significantly different (*P* < 0.01 and *P* < 0.001, respectively); value with “ns” above the bars indicated no significantly different. **(B–D)** Whole mount *in situ* hybridization analysis of *sema6D* in zebrafish embryos at different stages, blue arrowheads indicate blood vessels. **(E)** The procedure of the endothelial cells sorting and RT-PCR. **(F)** The agarose gel electrophoresis results of RT-PCR on *fli1a-EGFP* sorted cells.

To explore the roles of *sema6D* during zebrafish embryonic development, the expression level of *sema6D* was studied using QRT-PCR and WISH. According to QRT-PCR results, *sema6D* exhibited steady expression from 24 hpf to 96 hpf, with the highest level at 72 hpf, followed by 96 hpf (Figure 2A). Then, the spatial expression pattern of *sema6D* in early embryonic development was further studied by WISH. From 24 hpf to 48 hpf, *sema6D* was mainly expressed in the nervous system and also in the blood vessels (Figures 2B–D). Its expression in the nervous system is much restricted to the brain and head structures, being very weak in the spinal cord. Its expression in the vascular system was mainly observed in the ISVs. To further analyze the expression of *sema6D* in the zebrafish vessels, the endothelial cells from *Tg(fli1a:EGFP)* were sorted for RT-PCR (Figure 2E). The results showed that both fli1a and *sema6D* were detected in the selected endothelial cells, which was consistent with the previously reported single-cell RNA sequencing data of zebrafish endothelial cells (Shi et al., 2020; Figure 2F; Supplementary Table S1). Besides, mef2aa, which is specifically expressed in somite and heart, was chosen as a negative control to validate the purity of selected cells (Lv et al., 2017). The results showed that no mef2aa signals were detected in the sorted EGFP-positive cells (Figure 2F). Taken together, these results suggested that *sema6D* might participate in the development of zebrafish vessels and nerves.

### Deficiency of *sema6D* Caused Developmental Defect of PMNs

**Figure 3:**
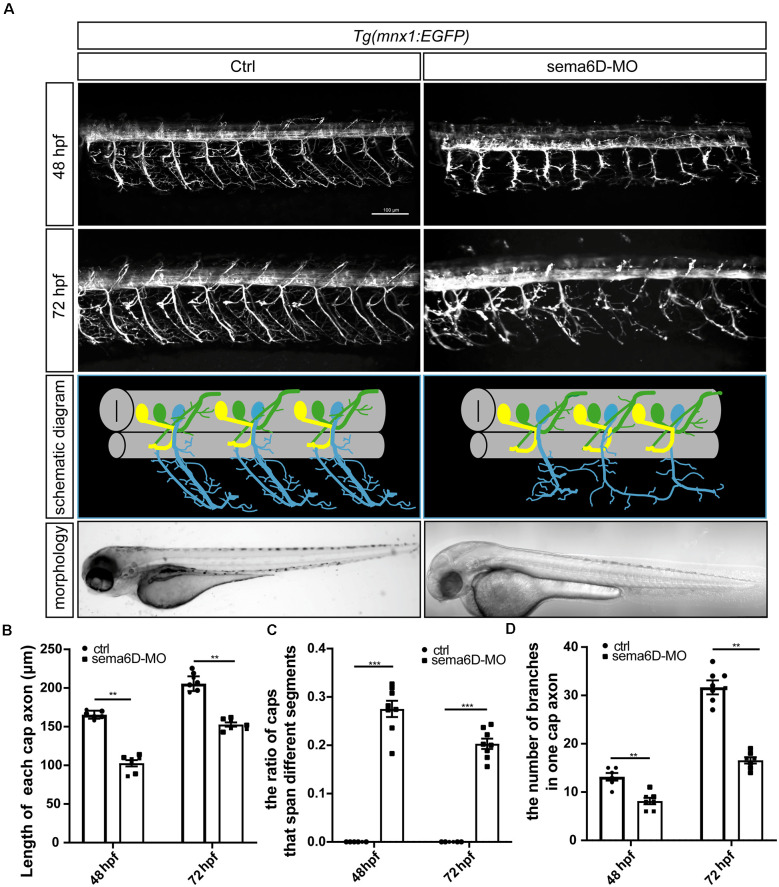
Primary motor neuron morphogenesis defects in the *sema6D* knockout zebrafish. **(A)** Confocal imaging analysis of PMNs in control and *sema6D* knockout groups at 48 and 72 hpf. **(B)** The statistical analysis of the length of each cap axon in the control and *sema6D* morphants at 48 and 72 hpf (*n* = 7). The length of five cap axon was measured in each zebrafish and the average was used. Mann-Whitney test, *P* = 0.0012. Values with ** above the bars are significantly different (*P* < 0.01). **(C)** The statistical analysis of the ratio of Caps across different segments in the control and *sema6D* morphants at 48 and 72 hpf (*n* = 8). Mann-Whitney test, *P* = 0.0007. Values with *** above the bars are significantly different (*P* < 0.001). **(D)** The statistical analysis of the number of branches in one cap axon in the control and *sema6D* morphants at 48 and 72 hpf. The number of branches in each cap axon was measured in five axon of each zebrafish and the average was used. Mann-Whitney test, 48 hpf: *P* = 0.0017; 72 hpf: *P* = 0.0012. Values with ** above the bars are significantly different (*P* < 0.01).

Considering the significant expression of *sema6D* in the zebrafish brain, it is rational to speculate it might modulate the development of neural system. To validate the hypothesis, specific morpholino antisense oligonucleotide (*sema6D*-MO) was used to knockdown the expression of *sema4D* in *Tg(mnx1:EGFP)* transgenic zebrafish. The results of cDNA amplification provided evidence that the injection of *sema6D*-MO efficiently altered the transcription pattern of *sema6D* (Supplementary Figure S1). Themorphology of PMNs was examined by confocal microscopy at 48 and 72 hpf. Although the morphant zebrafish embryos grossly appeared normal, the deficiency of *sema6D* caused dramatic developmental defects of PMNs (Figure 3A). Compared with the controls, the development of caudal primary motor neurons (Caps) was significantly inhibited at 48 hpf, where the average length of CaPs in the *sema6D* morphants was particularly shorter than that of the control groups (Figure 3B). At 72 hpf, although the length of CaP in the morphants were similar to the controls, the axonal trajectories of PMNs were significantly misled and many of the truncated axons in the *sema6D* deficient zebrafish could not recover completely (Figure 3C). Moreover, the number of CaP branches in the morphants decreased obviously at 72 hpf (Figure 3D). Taken together, these results suggest that *sema6D* is required for the development and navigation of neural networks.

### Deficiency of *sema6D* Caused Developmental Defects of Vascular Pattern

**Figure 4:**
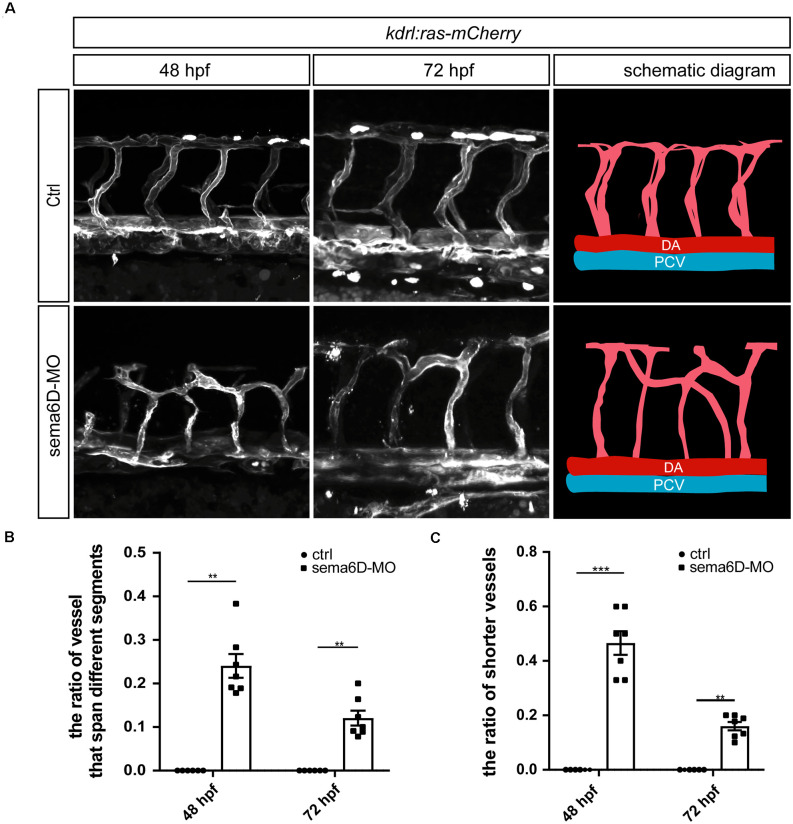
Deficiency of *sema6D* caused aberrant vascular networks. **(A)** Confocal imaging analysis of ISVs in the control and *sema6D*-MO embryos at 48 and 72 hpf. **(B)** The statistical analysis of the ratio of vessels that span different segments in the control and *sema6D* morphants at 48 and 72 hpf (*n* = 7). About 9–12 ISVs were used for statistics per zebrafish. Mann-Whitney test, *P* = 0.0012.Values with ** above the bars are significantly different (*P* < 0.01).**(C)** The statistical analysis of the ratio of shorter vessels in the control and *sema6D* morphants at 48 and 72 hpf (*n* = 7). About 9–12 ISVs were used for statistics per zebrafish. Mann-Whitney test, 48 hpf: *P* = 0.0006; 72 hpf: *P* = 0.0012. Values with ** and *** above the bars are significantly different (*P* < 0.01 and *P* < 0.001, respectively).

Since *sema6D* was found to express in zebrafish vessels, we speculated that it might participate in the development of blood vessel. To further explore the roles of *sema6D* in blood vessel formation, the morphology of ISVs in *sema6D* knockdown zebrafish was observed by confocal microscopy at different stages. The results showed that the deficiency of *sema6D* resulted in significant path finding defects of zebrafish ISVs, which was consistent with observation in the development of motor neuron. In control groups, ISVs grew from the dorsal aorta and reached the dorsal roof in an orderly manner to form dorsal anastomotic vessels (DLAV). In contrast, the ISVsin *sema6D* deficiency zebrafish grew upwards halfway, then turned to connect with adjacent ISVs disorderly, and could not form complete DLAV (Figures 4A,B). In addition, a small number of ISVs which grew only halfway or even less usually failed to cross the horizontal myoseptum in the *sema6D* knockdown zebrafish (Figure 4C). These results indicated that *sema6D* seems not necessary for the initial stages of ISVs sprouting, but rather regulates the vascular patterning.

### Knockout of *sema6D* Caused Aberrant Patterns of Both Nerves and Vascular System

**Figure 5:**
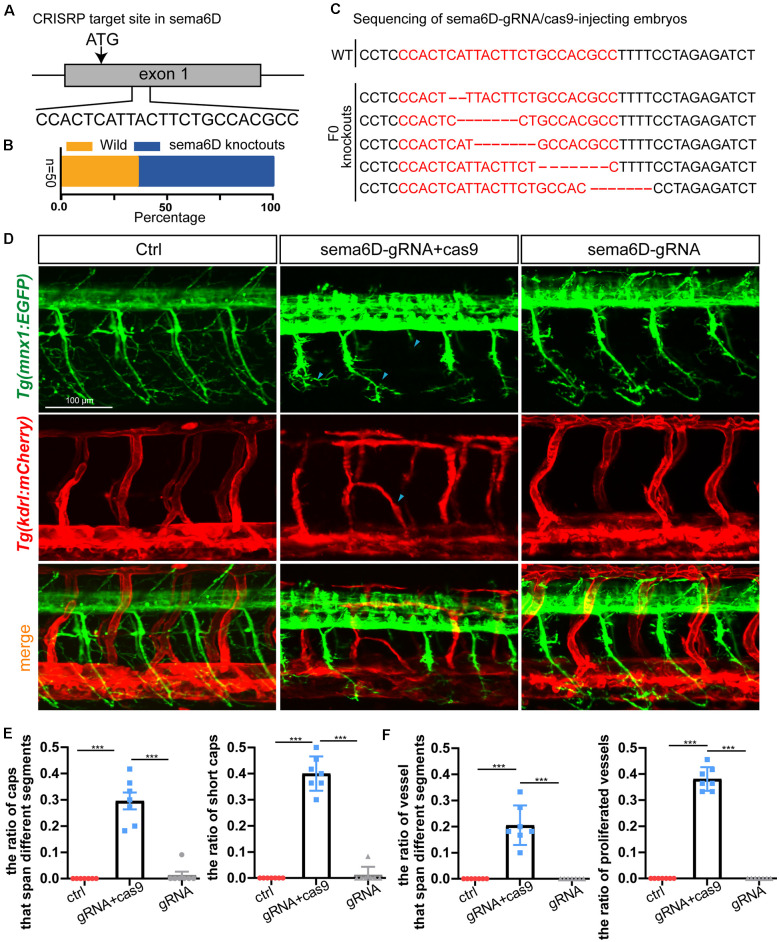
Knockout of *sema6D* caused both aberrant phenotypes in PMNs and ISVs. **(A)** Schematic diagram showing the targeting site of the gRNA on the *sema6D* gene. Starting codon (ATG) site is indicated by arrow. **(B)** The statistics of the ratio of wildtype to mutant sequences (*n* = 50). **(C)** Knockout patterns of *sema6D*-gRNA/cas9-injecting embryos by sequencing. **(D)** Confocal imaging analysis of primary motor neurons and intersegmental vessels in control and the F0 generation of the injected *Tg(mnx1:EGFP::kdrl:ras-mCherry)* zebrafish at 48 hpf, blue arrowheads indicate aberrant PMN and ISV. **(E)** The statistical analysis of the ratio of aberrant axonal projection of Caps (*P* = 0.0006) and short Caps (*P* = 0.0006) in the wild, F0 knockouts, and cas9 negative control at 48 hpf (*n* = 7). About 9–11caps were used for statistics per zebrafish. Mann-Whitney test. Values with *** above the bars are significantly different (*P* < 0.001). **(F)** The statistical analysis of the ratio of vessels that span different segments (*P* = 0.0006) and the ratio of proliferated vessels (*P* = 0.006) in the wild, F0 knockouts, and cas9 negative control at 48 hpf (*n* = 7). About 9–12 ISVs were used for statistics per zebrafish. Mann-Whitney test. Values with *** above the bars are significantly different(*P* < 0.001).

In order to confirm that *sema6D* is required for the development of PMNs and ISVs, the CRISPR/Cas9 system was utilized to knockout *sema6D* in *Tg(mnx1:EGFP::kdrl:ras-mCherry)* transgenic zebrafish line. In order to ensure complete disruption of functional proteins, the target sites near and downstream of the translation start codon (ATG) of *sema6D* coding sequence were selected for gRNAs design (Figure 5A). The editing efficiency and the knockout patterns of the selected gRNA-Cas9 system were identified by sequencing (Figures 5B,C). It was found that the phenotypes of PMNs and ISVs in the F0 generation of *sema6D* knockouts were consistent with the deficient morphants by confocal imaging analysis (Figure 5D). The PMNs were shorter and the axonal trajectories were apparently misled in the F0 knockouts (Figure 5E). Besides, the disorganized vasculature was also observed (Figure 5F). Furthermore, injection of *sema6D* gRNA without cas9 caused no obvious developmental defects, confirming the phenotype was a specific consequence of *sema6D* knockdown.

### Overexpressing *sema6D* Partially Restored the Defects of ISVs and PMNs in *sema6D* Deficient Embryos

**Figure 6:**
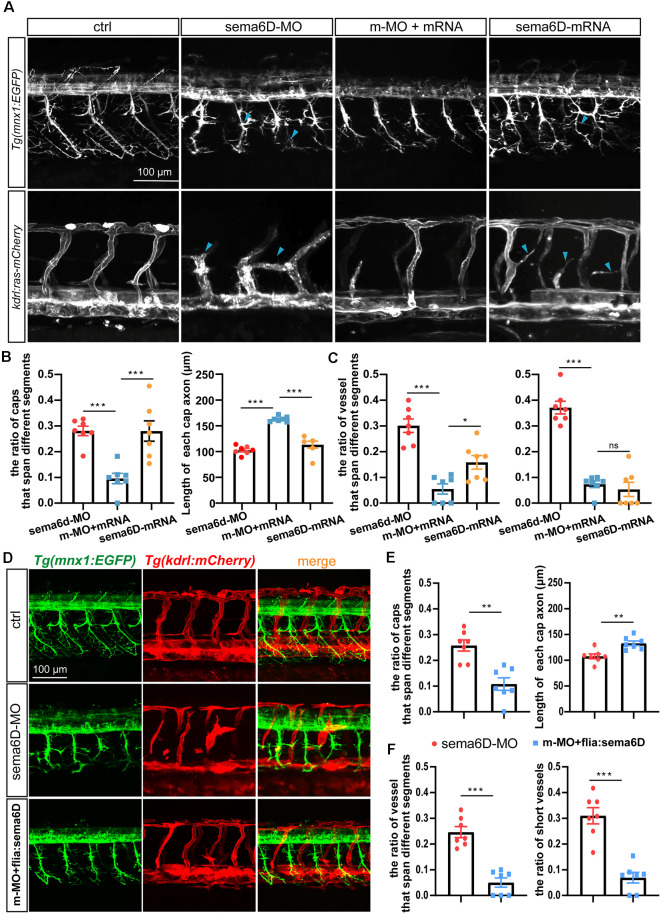
Overexpressing *sema6D* partially restored the defects of PMNs and ISVs in *sema6D*-deficient embryos. **(A)** Confocal imaging analysis of PMNs and ISVs in control, *sema6D*-MO, and *sema6D*-mRNA injected embryos at 48 hpf. Blue arrowheads indicate aberrant PMNs and ISVs. **(B)** The statistical analysis of the ratio of aberrant axonal projection of Caps (*P* = 0.0006) and short Caps (*P* = 0.0006) in *sema6D*-MO, m-MO+*sema6D*-mRNA, and *sema6D*-mRNA injected embryos at 48 hpf (*n* = 7). About 9–11 caps were used for statistics per zebrafish. Mann-Whitney test. Values with *** above the bars are significantly different (*P* < 0.001). **(C)** The statistical analysis of the ratio of vessel that span different segments (***P = 0.0006; *P = 0.0291) and the ratio of ectopic vessels (*P* = 0.0006) in the *sema6D*-MO, m-MO+*sema6D*-mRNA, and *sema6D*-mRNA injected embryos at 48 hpf (*n* = 7). About 9–12 ISVs were used for statistics per zebrafish. Mann-Whitney test. Values with *** above the bars are significantly different (*P* < 0.001). Value with “ns” above the bars indicated no significantly different. **(D)** Confocal imaging analysis of PMNs and ISVs in control, *sema6D* morphants, and *sema6D* morphants with *fli1a:sema6D* plasmid at 48 hpf. **(E)** The statistical analysis of the ratio of aberrant axonal projection of Caps (*P* = 0.0012) and the length of Caps (*P* = 0.0047) in the *sema6D* morphants and *sema6D* morphants with *fli1a:sema6D* plasmid at 48 hpf (*n* = 7). About 9–11 caps were used for statistics per zebrafish. Mann-Whitney test. Values with ** above the bars are significantly different (*P* < 0.01). **(F)** The statistical analysis of the ratio of vessels that span different segments (*P* = 0.0006) and the ratio of ectopic vessels (*P* = 0.0006) in the *sema6D* morphants and *sema6D* morphants with *fli1a:sema6D* plasmid at 48 hpf (*n* = 7). About 9–12 ISVs were used for statistics per zebrafish. Mann-Whitney test. Values with *** above the bars are significantly different (*P* < 0.001).

In order to confirm the defects of vascular and neuronal development were specifically caused by *sema6D* deficiency, the *in vitro* synthesized *sema6D* mRNA and *sema6D*-Mo were co-injected into one cell stage zebrafish embryos. Confocal imaging analysis revealed that the overexpression of *sema6D* mRNA could greatly rescue the defective phenotypes of PMNs and ISVs (Figures 6A–C) in *sema6D* deficient embryos. In addition, single *sema6D* mRNA injection also caused phenotypes of PMNs and ISVs, which are similar to those in deficient morphant (Figures 6A–C). This result validated that *sema6D* could regulate growing guidance of vessels and neurons in zebrafish. To further investigate the consequences of tissue specific rescue, *sema6D* was over-expressed in morphants with the fli1a promoter. Comparing with the morphant, embryos co-injected with *sema6D*-MO and *fli1a:sema6D* plasmid exhibited rescue phenotypes (Figures 6D–F). Taken together, these results suggested that endothelial-derived *sema6D* were involved in both neural and vascular development.

## Discussion

In this study, we found *sema6D* played a dual role in regulating vascular and neuronal patterning for the first time by using unique advantages of the zebrafish model. First, detailed expression analysis confirmed that *sema6D* is enriched in the neural system as well as blood vessels. Then, the knockdown and knockout of *sema6D* disturbed the pattern of both ISVs and PMNs, suggesting a requirement for *sema6D* in guiding endothelial cells and neurons during zebrafish embryonic development. Furthermore, overexpression of *sema6D* in whole embryos and endothelial cells both significantly relieved the aberrant phenotypes of vessels and nerves in sema6D morphants, validating that endothelial-derived *sema6D* is of great importance to the development and patterning of embryonic nervous and vascular system in zebrafish.

Semaphoring signaling was initially recognized in guiding axon growth (Chisholm and Tessier-Lavigne, 1999; Goodman et al., 1999). However, recent studies have focused its roles outside the neural system. Sema6D belongs to the semaphore in family, which exerts its function in either cell–cell interaction or a long distances manner by releasing its extracellular domains (SEMA+PSI domains) from the cell surface as a secreted cytokine (Toyofuku et al., 2004a, b; Peng et al., 2016). Thus, *sema6D* may perform diverse important functions during development processes. Furthermore, *sema6D* and its receptor Plexin-A1 were found to promote endocardial cells migration during heart development in combination with VEGFR2, suggesting it may function as a proangiogenic factor (Toyofuku et al., 2004a; Sun et al., 2019). However, there is so far no data on the regulation of vascular development by *sema6D*. Here, our WISH results showed that *sema6D* was not only expressed in the nervous system, but also in the ISVs, suggesting *sema6D* may participate in the vascular development of zebrafish. Our knock-down study provides first *in vivo* evidence supporting the expectation. The deficiency of *sema6D* resulted in abnormal ISVs patterning in zebrafish embryo. This result is consistent with previous studies, which have suggested that the organization of vascular system share various common guiding factors involved in nerves network (Sakurai et al., 2012; Zhang et al., 2020). However, there is another *sema6D* related gene in zebrafish, *sema6Dl*, whose function is unclear. Interestingly, our evolutionary analysis showed that *sema6Dl* did not cluster with *sema6D* in zebrafish but with other species, suggesting it may not be functionally related to *sema6D* in zebrafish. Furthermore, previous reports have found that *sema6Dl* was mainly expressed in the brain, lens, vagal ganglion, and retinal ganglion cells, but not in the primary motor neurons or the vascular system (Supplementary Table S1, Ebert et al., 2012; Shi et al., 2020). It is rational to speculate there is no redundant function between *sema6D* and *sema6Dl* in PMNs and ISVs development. Therefore, our data and previous studies remind us that *sema6D* might contribute to the endothelial cell formation and path finding.

Although *sema6D* has been validated in regulating specific axons projection as a guidance cue, its functional roles in animal motor neurons development remain unclear (Kimura et al., 2007; Kuwajima et al., 2012; Key et al., 2013). Here, our study demonstrated that *sema6D* is of great importance to the development of embryonic motor neurons in zebrafish. The deficiency of *sema6D* could lead to obvious motor neuron defects, including the inhibition of Caps growth and reduced branching of CaP axons. Meanwhile, the absence of* sema6D* caused dramatic aberrant patterning of PMNs, suggesting *sema6D* could regulate motor neurons path finding as a guidance signal. Interestingly, the previous report and our *in situ* hybridization results demonstrated that the expression of *sema6D* was not significant in the spinal cord (Kucenas et al., 2009). An important question is how does *sema6D* participate in motor neurons especially Cap axon guidance? From our results, the deficient phenotypes of PMNs are not always accompanied by abnormal ISVs, indicating the motor neurons defects are not the consequence of the aberrant vascular patterning. Another hypothesis is that *sema6D* acts as a guidance cue, which requires the receptors on motor neurons. Accordingly, a recent report demonstrated knockdown of plexin A1 leads to axons defect in somites, which is similar to our phenotypes (Dworschak et al., 2021). Furthermore, previous study in mouse also showed that developing embryonic but not mature adult blood vessels expressed Plexin-A1 andPlexin-A1 injected led a significant number of abnormal angiogenic spouts in zebrafish ISVs (Jacob et al., 2016). Therefore, our data and previous studies remind us that *sema6D* might contribute to the PMNs and ISVs patterning by binding to plexin A1 on motor neurons as well as endothelial cells. To further explore the relationship between the vascular and PMNs phenotypes, the tissue-specific rescue experiments were performed. The results showed that overexpressing *sema6D* in endothelial cells partially rescued the deficient phenotypes in the morphants, indicating endothelial-derived *sema6D* contributes to the development and patterning of embryonic nervous and vascular systems in zebrafish.

In summary, the present study identified the essential and multifunctional roles of *sema6D* during the embryonic nervous and vascular development. Deficiency of *sema6D* could cause deficient neuronal and vascular navigation. Our findings here provided new clues to the synergistically functional and molecular mechanism of *sema6D* underlying the nerves and blood vascular development. It is of great significance to completely dissect the axon guidance signaling network to understand how the nervous and blood vessel system are built up.

## Data Availability Statement

The original contributions presented in the study are included in the article/Supplementary Material, further inquiries can be directed to the corresponding author.

## Ethics Statement

The animal study was reviewed and approved by Administration Committee of Experimental Animals, Jiangsu Province, China.

## Author Contributions

DL supervised and designed this project. JS and DL wrote the manuscript and analyzed the data. JS, JX, and KG performed the experiments. All authors contributed to the article and approved the submitted version.

## Conflict of Interest

The authors declare that the research was conducted in the absence of any commercial or financial relationships that could be construed as a potential conflict of interest.

## Publisher’s Note

All claims expressed in this article are solely those of the authors and do not necessarily represent those of their affiliated organizations, or those of the publisher, the editors and the reviewers. Any product that may be evaluated in this article, or claim that may be made by its manufacturer, is not guaranteed or endorsed by the publisher.
