# Supplementary material for: Sema6D Regulates Zebrafish Vascular Patterning and Motor Neuronal Axon Growth in Spinal Cord
Source: Front Mol Neurosci. 2022 Apr 7;15:854556. doi: 10.3389/fnmol.2022.854556 (PMC9021825; doi:10.3389/fnmol.2022.854556)
Supplement: Supplementary file 1 [file Data_Sheet_1.docx]

**-Supplementary data**


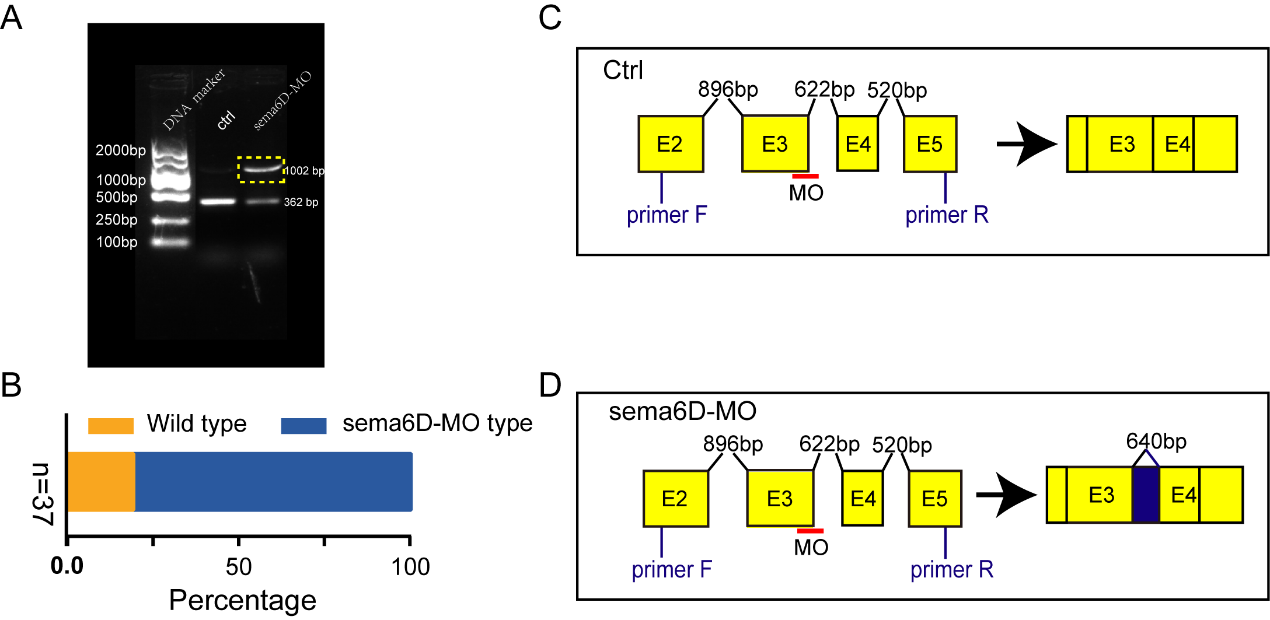


Figure S1. Sema6D-MO effectively reduced the expression of Sema6D through splice blocking. A. RT-PCR analysis of sema6D on AB embryos and sema6D-MO injected embryos; B. The pheno-typic statistics of knockdown efficiency (n=37); C. The schematic diagram of sema6D transcripts in wild embryos; D. The schematic diagram of sema6D transcripts in sema6D-MO injected embryos.
